# SGLT2 inhibitors as a novel senotherapeutic approach

**DOI:** 10.1038/s41514-025-00227-y

**Published:** 2025-05-10

**Authors:** Zeynep Elif Yesilyurt-Dirican, Ce Qi, Yi-Chian Wang, Annika Simm, Laura Deelen, Alia Hafiz Abbas Gasim, Fiona Lewis-McDougall, Georgina M. Ellison-Hughes

**Affiliations:** 1 Department of Pharmacology, Faculty of Pharmacy, Gazi University, Ankara, Türkiye; 2 School of Basic and Medical Biosciences, Faculty of Life Sciences & Medicine, Guy’s Campus, King’s College London, London, SE1 1UL UK; 3 Department of Life Sciences, Faculty of Natural Sciences, Imperial College London, London, UK; 4 Centre for Microvascular Research, William Harvey Research Institute, Faculty of Medicine and Dentistry, Queen Mary University of London, London, EC1M 6BQ UK

**Keywords:** Senescence, Endocrine system and metabolic diseases, Ageing

## Abstract

Cellular senescence is the permanent cessation of cell proliferation and growth. Senescent cells accumulating in tissues and organs with aging contribute to many chronic diseases, mainly through the secretion of a pro-inflammatory senescence-associated secretory phenotype (SASP). Senotherapeutic (senolytic or senomorphic) strategies targeting senescent cells or/and their SASP are being developed to prolong healthy lifespan and treat age-related pathologies. Sodium-glucose co-transporter 2 (SGLT2) inhibitors are a new class of anti-diabetic drugs that promote the renal excretion of glucose, resulting in lower blood glucose levels. Beyond their glucose-lowering effects, SGLT2 inhibitors have demonstrated protective effects against cardiovascular and renal events. Moreover, SGLT2 inhibitors have recently been associated with the inhibition of cell senescence, making them a promising therapeutic approach for targeting senescence and aging. This review examines the latest research on the senotherapeutic potential of SGLT2 inhibitors.

## Introduction

In the 1960s, Hayflick and Moorhead demonstrated that human fibroblasts have a limited ability to proliferate^1^. This phenomenon, known as replicative senescence, is associated with telomere shortening^2^ or damage and the activation of cell cycle inhibitors, such as p53/p21^CIP1/WAF1^ or p16^INK4a/Rb^^3,4^. Senescence plays an important role in the regulation of processes, such as tumour suppression, embryonic development and wound healing. It can be induced by oncogene activation, oxidative stress, radiation or epigenetic alterations in addition to telomere damage^5^. Senescent cells remain metabolically active and do not undergo apoptosis^6^. This ‘zombie cell’ state is characterised by the secretion of proinflammatory cytokines, growth factors, and proteases, known as the senescence-associated secretory phenotype (SASP)^7^, which exhibits both autocrine and paracrine actions^8^. Senescent cells create a pro-inflammatory microenvironment through their SASP, which can have a deleterious effect on neighbouring cells^9^. During aging, senescent cells accumulate in tissues and organs, which contributes to chronic disorders, including cardiovascular disease, diabetes, cancer, and metabolic syndrome^3,5^.

Sodium-glucose co-transporter 2 (SGLT2) inhibitors are a recent class of antidiabetic drugs that target SGLT2 located in the renal proximal tubules. SGLT2 is responsible for around 80–90% of renal glucose reabsorption, and SGLT2 inhibitors reduce blood glucose levels by suppressing renal glucose reabsorption^10^. Beyond their anti-diabetic effect, SGLT2 inhibitors have shown beneficial effects on major adverse cardiovascular events and improved outcomes in chronic kidney disease. The EMPA-REG OUTCOME trial reported a reduction in major cardiovascular disease events in empagliflozin-treated type 2 diabetic patients. In addition, the risk of hospitalisation for heart failure was 35% lower in patients treated with empagliflozin^11,12^. Moreover, empagliflozin showed improved cognitive impairment in older frail patients with type 2 diabetes and heart failure with preserved ejection fraction (HFpEF)^13^. Similarly, the CANVAS and DECLARE-TIMI 58 clinical trials demonstrated that canagliflozin and dapagliflozin reduced the risk of cardiovascular death or hospitalisation for heart failure in individuals with type 2 diabetes and cardiovascular disease, respectively^14–16^. The DELIVER trial suggests that the improvements due to dapaglifozin are increased the greater degree of frailty^17,18^. In patients with chronic kidney disease, regardless of their diabetes status, SGLT2 inhibitors (i.e. dapagliflozin) reduce the risk of death due to renal or cardiovascular causes^19,20^. The beneficial effects of SGLT2 inhibitors are likely to be independent of glycemic control, and the mechanisms behind these effects have been extensively debated^21–23^.

Although the mechanisms of the cardiovascular and renal benefits of SGLT2 inhibitors are not yet fully understood, several hypotheses have been proposed. It is suggested that reducing body weight and blood pressure^24^, inhibiting Na^+^/H^+^ exchanger^25^, reducing inflammation^26^ and oxidative stress^27^ may contribute to their efficacy^28^. Furthermore, SGLT2 inhibitors lead to a net loss of calories by promoting glucose excretion through urine. This metabolic alteration stimulates the utilisation of ketones and fatty acids^29^.

Senotherapeutics (senolytics and senomorphics) aim to eliminate or inhibit senescent cells and/or the deleterious effects of the SASP, making them a promising therapeutic approach in age-related diseases^3,30,31^. A recent review focusing on pharmacological modulation of vascular aging discussed promising senotherapeutic targets and the potential of some anti-diabetic drugs, including SGLT2 inhibitors, to reverse vascular aging^32^. Recently, SGLT2 inhibitors have been reported to suppress senescent cell burden^33,34^, and have been proposed as senomorphic drugs due to their ability to reduce inflammation^35^. Their use in age-related cardiovascular diseases has been recommended^36^. In this review, we summarise recent studies on SGLT2 inhibitors as potential senotherapeutics either with or without diabetes.

## Diabetes and senescence

**Fig. 1:**
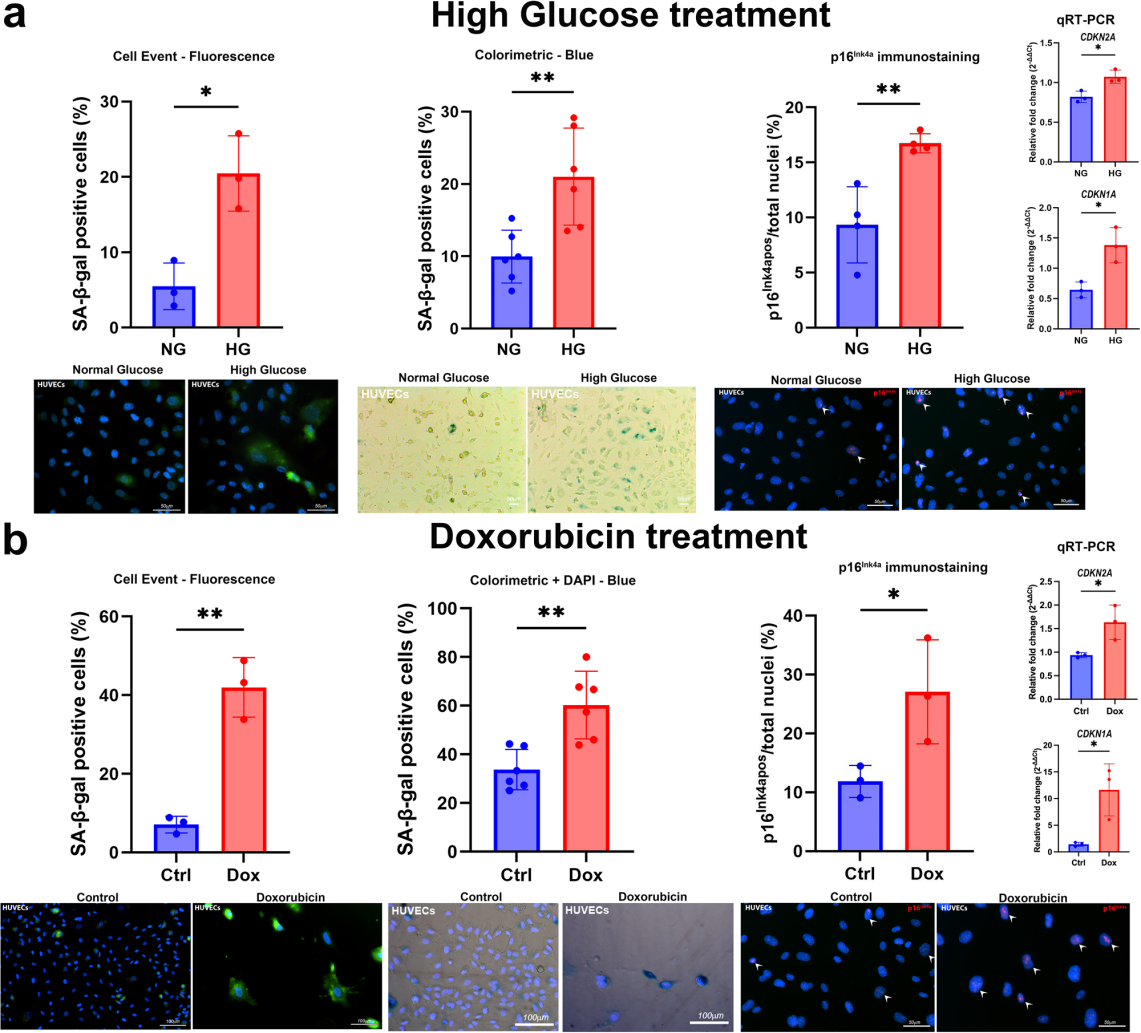
High glucose and doxorubicin treatment induces senescence to HUVECs. **a** HUVECs were treated for 6 days with high glucose (30 mM; HG) media or normal glucose (5.5 mM; NG) media. Senescence was determined by SA-β-gal (CellEvent™ Senescence Green Detection Kit (Thermo Fisher) and colorimetric Senescence β-Galactosidase Staining Kit #9860 (Cell Signalling), p16^Ink4a^ immunostaining (Anti-CDKN2A/p16INK4a antibody [EPR1473], Abcam, ab108349; p16^Ink4a^ positive cells indicated by arrowheads), qRT-PCR for *CDKN2A (p16)* and *CDKN1A (p21)*. **b** HUVECs were treated for 24 h with 0.2 µM doxorubicin and followed up for 3 or 7 days post-dox treatment. Senescence was determined by SA-β-gal (CellEvent™ Senescence Green Detection Kit (Thermo Fisher) and colorimetric Senescence β-Galactosidase Staining Kit #9860 (Cell Signalling) at 7 days post-dox. p16^Ink4a^ immunostaining (Anti-CDKN2A/p16INK4a antibody [EPR1473], Abcam, ab108349; p16^Ink4a^ positive cells indicated by arrowheads), and qRT-PCR for *CDKN2A (p16)* and *CDKN1A (p21)* was carried out at 3 days post-dox. Early passage HUVECs (Lonza) were cultured in Endothelial Cell Growth Medium (PromoCell, C-22010). HUVECs were grown at 37 °C in 5% CO_2_ and 19% O_2_. For quantification of SA-β-gal positive cells by CellEvent or p16^Ink4a^ positive cells, each well was imaged at ×20 magnification and ~10 FOV images were acquired per well. Using Image J, the number of SA-β-gal positive (green fluorescence) or p16^Ink4a^ positive (red fluorescence) cells were counted, and results were expressed as a percentage of total DAPI nuclei or cells. For quantification of SA-β-gal positive cells by colorimetric assay, each well was imaged at ×10 magnification and ~5 FOV images were acquired per well. Using Image J, the number of SA-β-gal positive (blue) cells were counted, and results were expressed as a percentage of total cells. In **b**, for the colorimetric assay, nuclei were counterstained using DAPI. Data are Mean ± SD. The data displayed normal variance. Significance between two groups was determined by Student’s *t* test using GraphPad Prism (GraphPad Software). *p* < 0.05 was considered significant. Each dot represents an individual well. *p < 0.05, **p < 0.01.

**Fig. 2:**
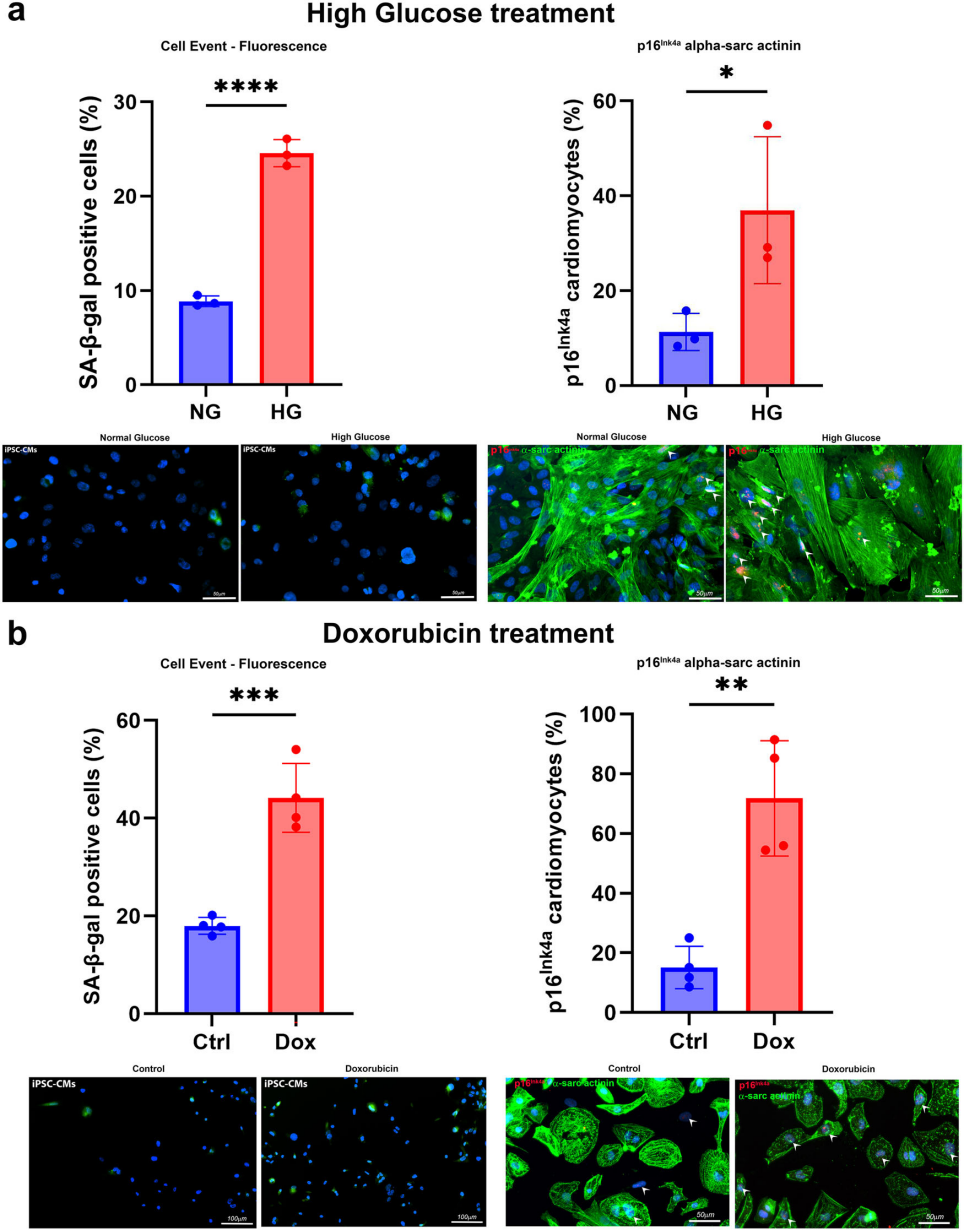
High glucose and doxorubicin treatment induces senescence to human iPSC-derived cardiomyocytes. **a** iPSC-derived cardiomyocytes were treated for 6 or 14 days with high glucose (30 mM; HG) media or normal glucose (5.5 mM; NG) media (STEMdiff™ Cardiomyocyte Maintenance Kit, #05020). Senescence was determined by SA-β-gal (CellEvent™ Senescence Green Detection Kit (Thermo Fisher)) after 6 days treatment, and p16^Ink4a^ immunostaining of cardiomyocytes (Anti-CDKN2A/p16INK4a antibody [EPR1473], Abcam, ab108349 and Anti-ACTN2 Antibody, Sigma, A7811; p16^Ink4a^ positive (red fluorescence) nuclei indicated by arrowheads) after 14 days treatment. **b** iPSC-derived cardiomyocytes were treated for 24 h with 0.2 µM doxorubicin and followed up for 14 or 21 days post-dox treatment. Senescence was determined by SA-β-gal (CellEvent™ Senescence Green Detection Kit (Thermo Fisher)) at 14 days post-dox treatment, and p16^Ink4a^ immunostaining of cardiomyocytes (Anti-CDKN2A/p16INK4a antibody [EPR1473], Abcam ab108349 and Anti-ACTN2 Antibody, Sigma, A7811; p16^Ink4a^ positive (red fluorescence) nuclei indicated by arrowheads) at 21 days post-dox treatment, Cardiomyocytes were sourced from Axol Bioscience (ax2508, Human iPSC-Derived Ventricular Cardiomyocytes (male)) or iPSCs were generated from neonatal male fibroblasts (AGFCL05) and differentiated to cardiomyocytes using Thermo Fisher cardiomyocyte differentiation media (A2921201, PSC Cardiomyocyte Differentiation Kit, Thermo Fisher Scientific (Life Technologies)). For quantification of SA-β-gal positive cells by CellEvent, each well was imaged at ×20 magnification and ~10 FOV images were acquired per well. Using Image J, the number of SA-β-gal positive (green fluorescence) cells were counted, and results were expressed as a percentage of total DAPI nuclei or cells. For quantification of p16^Ink4a^ positive cardiomyocytes, each well was imaged at ×40 magnification and ~10 FOV images were acquired per well. Using Image J, the number of p16^Ink4a^ positive (red fluorescence) ACTN2-positive cardiomyocytes (green fluorescence) were counted, and results were expressed as a percentage of total cardiomyocytes. Data are Mean ± SD. The data displayed normal variance. Significance between 2 groups was determined by Student’s *t* test using GraphPad Prism (GraphPad Software). *p* < 0.05 was considered significant. Each dot represents an individual well. *p < 0.05, **p < 0.01, ***p < 0.001, ****p < 0.0001.

Diabetes mellitus is a trigger of cellular senescence^37^ and is associated with age-related cardiovascular and renal diseases. However, the SASP may also contribute to the development of type 2 diabetes^38^. For this reason, type 2 diabetes has been hypothesised to be both a cause and a consequence of senescence^39^. Type 2 diabetes is associated with the accumulation of senescent cells, particularly insulin-producing pancreatic β cells^40^, and T cells^41^, and these senescent cells are important contributors to the onset and progression of many pathologies in diabetics. This proposed link between senescent cell burden and type 2 diabetes^42,43^ has also been demonstrated in cells exposed to high glucose in vitro. Indeed, hyperglycaemia has been reported to increase senescence markers in various cell types^44–46^, such as dermal-derived human microvascular endothelial cells, human umbilical vein endothelial cells (HUVECs) and human retinal microvascular endothelial cells (HRMECs), and is considered to be one of the triggers of senescence. It has been suggested that hyperglycaemia contributes to senescence induction by stimulating the production of reactive oxygen species (ROS), the accumulation of advanced glycation end products (AGEs) and DNA damage^47^. We show that treatment of HUVECs and human iPSC-derived cardiomyocytes with high glucose (30 mM) media leads to increased SA-β-gal, p21 and p16^Ink4a^ expression, compared to standard (5.5 mM) glucose media (Figs. 1 and 2). The expression profile of these senescent markers is comparable to other inducers of senescence, such as doxorubicin premature stress-induced senescence to HUVECs and human iPSC-derived cardiomyocytes (Figs. 1 and 2).

## SGLT2 inhibitors

Introduced as a new class of drugs for the treatment of type 2 diabetes, SGLT2 inhibitors (e.g., dapagliflozin, canagliflozin, empagliflozin) inhibit the reabsorption of glucose in the kidney, leading to glycosuria. In addition to their blood glucose lowering effect, several clinical trials have shown the advantages of SGLT2 inhibitors on renal and cardiovascular outcomes, with and without diabetes^11,19,22,48–54^. On the other hand, it is important to be aware of their potential adverse effects. Increased glucosuria may lead to urinary tract and genital infections^11,49,50^. Moreover, hypotension^50^ and hypovolemia risk^55^ could be observed as a consequence of natriuretic and blood-pressure lowering effects. A risk of ketoacidosis was also reported in some cases^15,52,53,56^. Despite the absence of robust evidence indicating an increased risk of hypoglycaemia^49^, it is acknowledged that the concomitant administration of insulin secretagogues potentially contributes to the occurrence of hypoglycemia^57,58^.

**Fig. 3:**
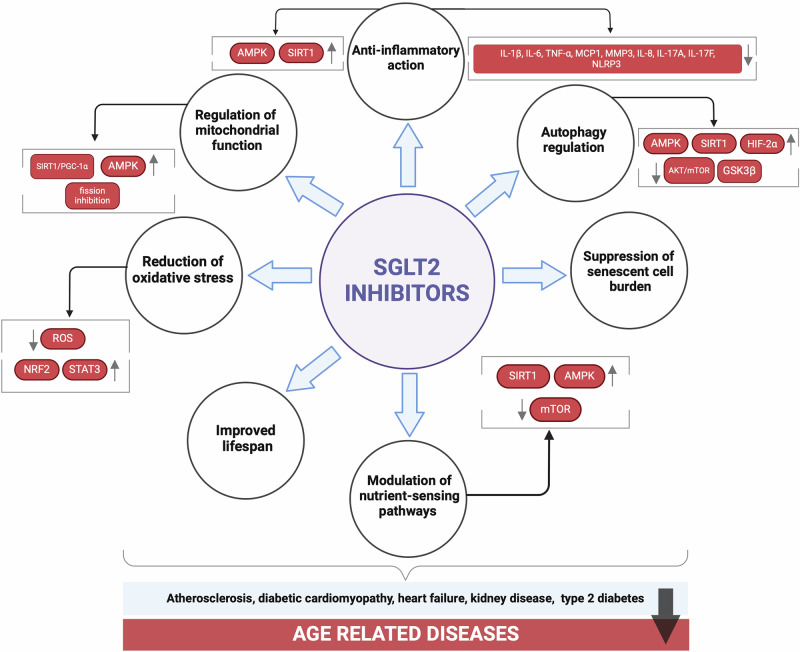
The anti-ageing actions of SGLT2 inhibitors. SGLT2 sodium-glucose co-transporter 2, mTOR Mammalian Target of Rapamycin, AMPK AMP-activated protein kinase, SIRT1 Sirtuin1, NRF2 nuclear factor erythroid 2-related factor 2, STAT signal transducer and activator of transcription, HIF-2α hypoxia-inducible factor-2α, GSK3β glycogen synthase kinase 3 beta. Generated with BioRender (https://biorender.com/).

Nevertheless, the recent attention on repositioning SGLT-2 inhibitors as senotherapeutic agents targeting aging is noteworthy. The anti-aging effects of SGLT2 inhibitors have been attributed to multiple mechanisms. These include the reduction of inflammation, free radicals, and oxidative stress; regulation of mitochondrial function and autophagy; modulation of nutrient-sensing pathways such as mammalian target of rapamycin (mTOR), AMP-activated protein kinase (AMPK), and sirtuins (SIRT); suppression of senescent cell burden; and extension of lifespan^29,34,35,59–61^ (Fig. 3). SGLT2 inhibitors have been suggested to mimic calorie restriction by increasing AMPK and SIRT1, while decreasing mTOR and insulin/IGF1 (insulin-like growth factor 1) signalling, which may potentially affect longevity^62^. Furthermore, increasing evidence points to the benefits of SGLT2 inhibitors through mTOR, AMPK and SIRT1 pathways in different tissues and cells, such as kidney^63^, cardiac microvascular endothelial cells^64^, and cardiomyocytes^65^. In this review, we focus on the effects of SGLT2 inhibitors in various models of senescence and their mechanism of action. Table 1 summarises the studies that show anti-senescence effects of SGLT2 inhibitors.

**Table 1:** Anti-senescence effects of SGLT2 inhibitors

Model	SGLT2 inhibitor	Findings and mechanism of action	Reference
**Endothelial senescence**			
H_2_O_2_-induced senescence in HUVECs	Dapagliflozin	↓ Senescence markers (SA-β-gal, p21 and p53 expression) ↑ eNOS phosphorylation, SIRT1 expression, and NAD^+^/NADH ratio	^74^
db/db mice	Dapagliflozin	↓ Senescence markers (SA-β-gal, p53 and p21) in aorta and circulating inflammatory factors (IL-8, IL-17A and IL-17F)	^77^
HG-induced endothelial senescence in HUVECs	Dapagliflozin	↓ Senescence markers (SA‐β‐gal, p21, p53 expression) ↑ SIRT1 and NAD^+^/NADH ratio	^77^
Cardiac microvascular endothelial cell senescence in STZ diabetic mice	Empagliflozin	↓ SA-β-gal positive cells Suppression of mitochondrial fission and activation of AMPK	^78^
Palmitic acid-induced senescence in HUVECs	Canagliflozin	↓ Senescence markers (SA-β-gal, p16, p53), DNA damage, SASP factors (IL-6, TNF-α and MMP3) Inhibiting ROS and p38/JNK activation	^84^
Porcine coronary artery endothelial cells exposed to high glucose	Empagliflozin	↓ Senescence markers (SA-β-gal, p16, p21)	^87^
Nanoplastic-induced senescence in porcine coronary artery endothelial cells	Enavogliflozin	↓ Senescence markers (SA‐β‐gal activity, p53, p31) and NADPH oxidase markers (Nox2 and p22^phox^)	^75^
Ponatinib- induced senescence in human aortic endothelial cells	Empagliflozin/dapagliflozin	↓ SA-β-gal activity ↑ Pro-angiogenic activity and autophagy	^89^
Angiotensin II- induced endothelial senescence in porcine coronary arteries	Empagliflozin	↓ Senescence markers (p53, p21 and p16) pro-atherothrombotic markers (VCAM-1 and MCP-1)	^90^
D-gal- induced senescence in HUVECs	Empagliflozin	↓ Senescence markers (SA‐β‐gal, decreased EdU-positive staining, p53, and p21) and NLRP3 and p-STAT3	^97^
**Cardiovascular senescence**			
HFD fed obese mice	Canagliflozin	Enhanced senolytic function by NK or T cells and AMPK signalling, downregulation of PD-L1	^34^
HG- induced cardiac stromal cell senescence and STZ-induced diabetic mice	Empagliflozin	↓ SA‐β‐gal positive cells, P-p38 ↑ pAkt ↓ Cardiac SA-β-gal activity	^108^
Control ZSF1 rats	Empagliflozin	Senescence markers (p53, p21, p16), VCAM-1, tissue factor, SGLT1 and SGLT2 expression ↑ eNOS in aortic inner curvature	^110^
**Arterial stiffness**			
Aged C57BL/6J mice	Empagliflozin	F-actin and P-cofilin content in mesenteric arteries Arterial stiffening Downregulation of ROS synthesis	^119^
Aged, non-diabetic C57BL6/J mice	Empagliflozin	↓ Medial collagen type I TGF-β Aortic stiffness	^120^
**Renal senescence**			
Renal tubular epithelial cell senescence induced by HG	Dapagliflozin	↓ ROS production, DNA damage (γ-H2AX, pATM/ATM, p-p53/p53), senescence markers and SASP (IL-1, IL-8, and TGF-β1)	^127^
Renal proximal tubule cells from hedgehog interacting protein transgenic mice	Canagliflozin	↓ SA-β-gal activity	^128^
Type 2 diabetic mice	Dapagliflozin	↓ Senescence markers (p21, p16, and p53), DNA damage (γH2AX), SASP factors (MIP-1γ, MIP-2, and CXCL-related cytokines) in kidney ↑ Ketone body induced NRF2 activation AMPK, SIRT1, and PGC-1α	^33^
D-gal- induced renal senescence and HG-induced senescent HK2 cells	Empagliflozin	↓ SA-β-gal, p16, LCN2, and TNF-α	^153^
**β-cell senescence**			
HFD-induced obese mice	Dapagliflozin (with lobeglitazone)	↓ p16-positive cells in pancreas	^133^
**Stem and progenitor cell aging**			
Human bone marrow mesenchymal stem cells	Empagliflozin	↓ Senescence markers (SA‐β‐gal, p21) ↑ Cell proliferation and migration	^137^
**Adipose tissue and liver**			
Hereditary hypertriglyceridemic rats	Empagliflozin	↓ p21^Waf1^ in the liver and white adipose tissue	^144^
db/db mice	TA-1887	↓ IL-6, IL-1b, MCP1, and p16^INK4a^ in epididymal white adipose tissue	^60^
Naturally aged male C57BL/6J mice	Empagliflozin	Delayed liver senescence Upregulation of PI3K/AKT and AMPK/SIRT1 Downregulation of NF-κB	^61^
**Brain and neuro**			
D-gal- induced aging in C57BL/6J mice	Dapagliflozin	↑ Improved learning skills of aged mice SOD, SIRT1 and PGC-1α in brain tissue	^150^
**Lifespan**			
Premature aging mice model (Zmpste24 knockout)	Canagliflozin	Extended lifespan	^34^
7 months aged male mice	Canagliflozin	Extended median survival	^59^
Naturally aged male C57BL/6J mice	Empagliflozin	Improved lifespan and health	^61^

## The effects of SGLT2 inhibitors on cellular senescence

### Endothelial senescence

Senescent endothelial cells lead to endothelial dysfunction, impair vascular function and predispose to cardiovascular diseases. Senescence is also considered a trigger for endothelial dysfunction observed in diabetes. Indeed, endothelial cell senescence develops in the diabetic rat aorta^66^ and an increase in the expression of senescence-associated β-galactosidase (SA-β-gal) and p16^Ink4a^ has been reported in endothelial cells exposed to high concentrations of glucose^67,68^ (Fig. 1). It has been reported that high glucose promotes endothelial cell senescence through ROS mediated by superoxide dismutase (SOD) and SIRT as well as increased pro-inflammatory cytokines^3,69^. Increased proinflammatory cytokines like IL-1β, IL-6 and tumour necrosis factor-α (TNF- α), observed in plasma samples from COVID-19 patients, led to an upregulation of SGLT2 expression in endothelial cells, resulting in endothelial dysfunction and senescence^70^. Furthermore, the same authors have recently demonstrated that SGLT2 expression is associated with levels of low-grade inflammation and oxidative stress in the internal thoracic artery and left ventricle of patients undergoing bypass and aortic valve surgery, respectively^71^. In addition, increased pro-inflammatory cytokines have been observed to increase SGLT2 expression in porcine coronary endothelial cells, thereby promoting endothelial dysfunction and potentially leading to senescence^71^. Of note, it is important to emphasise that the baseline expression of SGLT2 in endothelial cells remains relatively low compared to renal proximal tubules, and the physiological and pathological role of SGLT2 expression in endothelial cells is not yet fully understood.

Endothelial senescence is associated with reduced endothelial nitric oxide synthase (eNOS)-mediated nitric oxide (NO) production^72^ and oxidative stress^73^. SGLT2 inhibitors have been reported to increase endothelium-derived NO bioavailability and improve vasodilatation in db/db mice^74^. Although it is conceivable that this effect may be associated with their glucose-lowering activity, the acute effect of dapagliflozin was also investigated in aortic rings of C57BL/6J mice and vasodilation was observed^74^. In addition, dapagliflozin treatment was found to reverse the decrease in NO levels in oxidative stress induced HUVECs^74^. Besides, two different SGLT2 inhibitors (dapagliflozin and empagliflozin) were reported to restore NO bioavailability in TNF-α induced inflammation model of human coronary arterial endothelial cells^27^.

SGLT2 inhibitors also have regulatory effects on endothelial cell proliferation^75^, migration and differentiation^76^. In addition, they have been shown to ameliorate endothelial cell senescence through their anti-inflammatory effects and suppression of ROS, and these protective effects have been associated with the activation of AMPK and SIRT1 signalling^74,77,78^. It is well established that AMPK and SIRT1 play a crucial role in the vasculature by reducing inflammation and oxidative stress^79,80^, and SGLT2 inhibitors could modulate these pathways in vascular aging. A recent study indicated that dapagliflozin reduced apoptosis, ROS and inflammation in high glucose-induced endothelial dysfunction by regulating the AMPK/SIRT1 pathway^81^. The reduction of inflammatory cytokine and chemokine secretion with another SGLT2 inhibitor, canagliflozin, in vascular endothelial cells was also mediated through an AMPK-dependent mechanism^82^. Moreover, it was reported that dapagliflozin treatment increased cell viability, suppressed elevated inflammatory factors (IL-1β, IL-6 and TNF-α), and induced autophagy by inhibiting the protein kinase B (AKT)/mTOR signalling pathway in HUVECs exposed to high glucose^83^. Another study by Hao et al.^84^ demonstrated that canagliflozin could attenuate palmitic acid-induced senescence in HUVECs, as well as inflammatory factors, such as IL-6 and TNF-α and MMP3 (matrix metalloproteinase 3) present in the SASP. Moreover, these effects were associated with the inhibition of ROS and p38/JNK (c-Jun N-terminal kinase) pathway. The mitogen-activated protein kinases (MAPKs), p38/JNK, have been reported to be involved in the regulation of cellular senescence^85,86^.

In a study investigating the possible role of SGLT2 in endothelial senescence and dysfunction, it was found that increased senescence markers (SA-β-gal, p16, p21 but not p53) in porcine coronary artery endothelial cells exposed to high glucose were inhibited by empagliflozin treatment^87^. Based on the relationship between oxidative stress and endothelial senescence, cells were also exposed to H_2_O_2_, however, no effect of empagliflozin on increased SA-β-gal activity was observed^87^. The potential effects of dapagliflozin on vascular senescence were investigated in another study. SA-β-gal, p53 and p21 were found to be increased in the aortas of type 2 diabetic mice compared to controls. Furthermore, the secretion of inflammatory factors such as IL-8, IL-17A and IL-17F was significantly increased in type 2 diabetic mice, and these effects were attenuated by dapagliflozin treatment^77^. Another study investigated the effect of enavogliflozin, which is currently undergoing clinical trials for type 2 diabetes, on endothelial senescence caused by nanoplastics. Nanoplastics induced cell cycle arrest by increasing the expression of senescence markers p53 and p21 and NADPH oxidase-mediated ROS generation in porcine coronary artery and endothelial cells. Inhibition of SGLT2 using enavogliflozin ameliorated the nanoplastic senescence-inducing effects. It is noteworthy that nanoplastics increased the expression of SGLT2 but not SGLT1 in these cells, suggesting a link between SGLT2 and nanoplastic-induced endothelial senescence^75^.

Ponatinib, a tyrosine kinase inhibitor, induces vascular senescence^88^. Interestingly, it was found to increase SGLT2 expression, decrease cell viability, and increase SA-β-gal activity in human aortic endothelial cells^89^. Furthermore, ponatinib inhibited endothelial tube formation. Both empagliflozin and dapagliflozin treatments were found to ameliorate ponatinib-induced endothelial senescence and induce pro-angiogenic activity. Additionally, empagliflozin was reported to induce autophagy in cells exposed to ponatinib^89^.

The angiotensin (Ang) system is an important trigger of endothelial dysfunction associated with aging and major cardiovascular diseases^90^. In endothelial cells isolated from porcine coronary arteries, Ang II was shown to increase SGLT1 and SGLT2 protein levels and SA-β-gal activity, all of which were inhibited by SGLT1 and 2 inhibitors, sotagliflozin and empagliflozin respectively. In addition, Ang II upregulated p53, p21 and p16 protein expression, which was abolished by both treatments. Ang II also downregulated eNOS protein expression and increased the pro-atherothrombotic markers, VCAM-1 and MCP-1, in endothelial cells, which were prevented by SGLT1 and 2 inhibitors^90^. Based on the induction of endothelial senescence by Ang II^91^, the role of the Ang system in high glucose-induced endothelial senescence and the effects of empagliflozin were also investigated. Protein expression of ACE and AT1 receptors was found to be increased by high glucose, while empagliflozin treatment decreased their expression. Furthermore, it was observed that high glucose levels led to an increase in SGLT1 and SGLT2 protein levels in cultured endothelial cells and endothelium. Inhibition of the Ang system prevented high glucose-induced senescence and the subsequent increase in SGLT1 and SGLT2 expression^87^.

D-galactose (D-gal) has been used in several studies to create an aging model in mice and rats that mimics natural aging^92–94^. Moreover, D-gal has been utilised in studies to induce senescence and investigate anti-aging treatments^95,96^. SGLT2 inhibitors have been shown to be effective against D-gal-induced cellular senescence. In HUVECs, empagliflozin significantly inhibited the increase of SGLT2, p53, and p21 protein expressions caused by D-gal. Empagliflozin treatment normalised the decrease in the percentage of EdU-positive cells and ameliorated SA-β-gal activity observed in D-gal-treated HUVECs. Furthermore, empagliflozin treatment reduced the elevated inflammatory molecule, NLRP3 in D-gal-treated HUVECs^97^.

Mitochondrial impairment is a common feature of aging and age-related pathologies^29,98^. Cardiovascular benefits of SGLT2 inhibitors have been attributed to improvements in mitochondrial function^99^ and their ability to modulate mitochondrial dynamics^100,101^. In a murine model of heart failure, empaglifozin lessened fibrosis in the heart by improving cardiac mitochondrial function^102^. Moreover, empagliflozin treatment was found to inhibit cardiac microvascular endothelial cell senescence in STZ-diabetic mice by suppressing mitochondrial fission through an AMPK-dependent pathway^78^.

### Cardiovascular senescence

Aging causes various structural and functional impairments in the cardiovascular system. With age, senescent cells accumulate in the heart and vasculature, leading to cardiovascular pathologies^103^. It has been shown that removing senescent cells in aged mice, either pharmacologically using senolytics or genetically, improved myocardial dysfunction, led to the formation of new cardiomyocytes and decreased hypertrophy and fibrosis associated with cardiac aging^104–106^. With age, the SGLT2 protein levels in cardiomyocytes increase. By inhibiting SGLT2, age-associated defects of the [Ca^2+^]_i_-homoeostasis, phospholamban (PLB) phosphorylation, Na^+^/Ca^2+^-exchanger (NCX) activity and mitochondrial Ca^2+^-loading can be restored^107^. Given their benefits on cardiovascular events and all-cause mortality, SGLT2 inhibitors may have anti-aging effects. In support of this, as reported by Katsuumi et al., the administration of canagliflozin to ApoE-knockout atherosclerotic mice contributed to the reduction of senescent cells and improvement of atherosclerosis^34^. In a study on high glucose-induced cardiac stromal cell senescence, empagliflozin treatment reversed the increase in SA-β-gal-positive cells and Phospho-p38, as well as the decrease in pAkt protein expression^108^. High glucose conditions disrupt the PI3K/Akt/eNOS pathway, leading to activation of mitogenic and pro-inflammatory factors^109^, therefore these findings highlight a possible link between downregulation of the PI3K/Akt pathway and senescence induction^108^. In another study investigating the cardiovascular effects of empagliflozin treatment in obese and lean control ZSF1 rats, the expression levels of senescence markers p53, p21 and p16 and SGLT1 and SGLT2 protein expression were increased in the inner aortic curvature, compared to the outer curvature in control lean rats, which was prevented by empagliflozin treatment^110^. In a murine diabetic cardiomyopathy model, treatment with empagliflozin attenuated apoptosis, fibrosis, autophagy, senescence and cardiac dysfunction. Furthermore, empagliflozin treatment inhibited hyperactivation of autophagy through the AMPK/GSK3β signalling pathway^111^. A recent study has also revealed that increased senescence markers (p53, p21, γ-H2AX, SASP and SA-β-Gal activity) in STZ and high fat diet (HFD)-induced type 2 diabetic mouse model and high glucose and palmitic acid-exposed AC16 cardiomyocyte cells were reversed by empagliflozin. The researchers have proposed a novel mechanism for the drug, suggesting that the amelioration of senescence was related to the suppression of the overexpressed ANGPTL4 and the regulation of the FOXO1 pathway^112^.

The increased expression of SASP proteins and senescence markers by cardiotoxic chemotherapeutic drugs like doxorubicin^113^ indicates a possible correlation between senescence, heart disease and cancer treatment. Doxorubicin, which increases the expression of p16^Ink4a^, p53/p21^Cip1/Waf1^ and SA-β-Gal activity in cardiomyocytes, has been linked to the development of cardiomyopathy^114^. A study proposing that doxorubicin-induced cardiotoxicity is associated with cardiomyocyte senescence, additionally noted beneficial effects on doxorubicin-induced cardiac dysfunction upon senolytic navitoclax treatment^115^. In a study investigating the effects of dapagliflozin on doxorubicin-induced cardiotoxicity, rats were given dapagliflozin for 6 weeks, followed by doxorubicin for 4 weeks. The group treated with doxorubicin exhibited a decrease in systolic function parameters, which were normalised by dapagliflozin pre-treatment. Additionally, in vitro studies demonstrated that dapagliflozin treatment reduced doxorubicin-induced apoptosis and normalised STAT3 expression in cardiomyocytes. Notably, the cardioprotective effect of dapagliflozin, suppressing doxorubicin-induced ROS and apoptosis, was shown to decrease when STAT3 was knocked down in cardiomyocytes^116^. This suggests that the cardioprotective effects of dapagliflozin are mediated by STAT3.

### Arterial stiffness

Vascular aging is associated with arterial stiffness^117^, and pathologies such as hypertension, diabetes and obesity accelerate the process of arterial stiffness. On the other hand, arterial stiffening is defined as an independent risk factor in the development of atherosclerosis^118^. SGLT2 inhibitors have been shown to alleviate age-related arterial stiffness. In an experiment with 80-week-old C57BL/6J male mice, empagliflozin treatment for 6 weeks resulted in decreased stiffness of the mesenteric artery and thoracic aorta compared to the control group. Mesenteric arteries from treated mice showed a reduction in F-actin content associated with arterial stiffening^119^. The study also revealed that empagliflozin treatment led to a downregulation of pathways involved in the synthesis of ROS in the aortas of mice, suggesting that the positive vascular effects of SGLT2 inhibition may be due to reduced oxidative stress^119^ In another study using aged, non-diabetic C57BL6/J mice, 7-week treatment with empagliflozin decreased age-related stiffness in the aorta. Additionally, the treatment resulted in a reduction of medial collagen type I and TGF-β levels in aged mice while having no impact on elastin fragmentation^120^. On the other hand, it has been suggested that empagliflozin’s positive effects on arterial stiffening may be due to the activation of specific receptor signalling pathways, independent of NO, inflammation or oxidative stress. In a study of patients with type 1 diabetes, empagliflozin, metformin, and a combination of both treatments showed comparable improvements in endothelial function. However, the empagliflozin/metformin treatment was found to improve arterial stiffness parameters superior to metformin alone. This implies that empagliflozin may affect arterial stiffness through a different mechanism, possibly involving SGLT2 receptors in vascular smooth muscle cells^121^. In clinical studies involving type 2 diabetics, blood pressure reduction with SGLT2 inhibitors has been associated with improvements in arterial stiffness and vascular resistance^122,123^. The mechanism of SGLT2 inhibitors on arterial stiffness is not yet fully understood, and further research is required to elucidate the underlying mechanisms.

### Renal senescence

Senescence is also associated with the development of diabetic nephropathy^124^ and senescent proximal tubular cells have been reported to contribute to the development of diabetic kidney disease^125^. Renal p21 expression was significantly increased in STZ-diabetic mice compared to control, while p16 and p27 expressions were similar. SA-β-gal staining was also increased in tubular epithelial cells of diabetic mice. The administration of low and high doses of insulin improved the diabetes-induced changes. On the other hand, the knockdown of p21 or SGLT2 reversed senescence induced by high glucose in human proximal tubular cells. These findings suggest that targeting SGLT2 and/or p21 could be a novel therapeutic approach to prevent renal senescence in diabetes^126^. Eleftheriadis et al.^127^ investigated the role of dapagliflozin in inhibiting senescence in renal tubular epithelial cells (RPTECs) exposed to high glucose. In primary RPTECs, high glucose led to an increase in SGLT2 expression and ROS production. Additionally, it caused DNA damage, as evidenced by increased γ-H2AX levels and increased SASP factors. Dapagliflozin treatment normalised these high glucose-induced changes, including decreasing the SASP factors, IL-1, IL-8, and TGF-β1^127^.

A study investigating the link between senescent renal tubular cells and hedgehog interacting protein in the development of tubulopathy associated with diabetic kidney disease, found that tubular senescence in STZ-diabetic mice was reduced when hedgehog interacting protein was knocked out in renal proximal tubule cells^128^. Interestingly, this reduction was associated with inhibition of SGLT2 expression, as SGLT2 expression was found to be reduced in knockout diabetic mice, compared to diabetic mice. In contrast, transgenic mice and HK-2 cells with overexpressed hedgehog interacting protein showed increased SGLT2 expression, which led to cell senescence. On the other hand, canagliflozin administration inhibited the increased SA β-gal activity in renal proximal tubule cells isolated from over-expressed hedgehog interacting protein transgenic mice^128^.

Another study compared the anti-senescent activities of the antidiabetic drugs glimepiride (a sulphonylurea) and dapagliflozin in the kidneys of type 2 diabetic mice. The mRNA and protein expression of p21, p16, and p53 increased in the kidneys of diabetic animals and was markedly reduced in the group receiving dapagliflozin but not glimepiride. Similar results were obtained for the DNA damage marker, γH2AX^33^. In addition, several SASP factors (MIP-1γ, MIP-2, and CXCL-related cytokines) that were increased in the kidneys of diabetic and glimepiride-treated diabetic groups were significantly decreased in dapagliflozin-treated diabetics. The study also examined whether the renoprotective effects of SGLT2 inhibitors were mediated by ketone bodies. For this purpose, HK-2 cells in which senescence was induced by H_2_O_2_ were pre-treated with β-hydroxybutyrate, and this resulted in the inhibition of senescence markers^33^. These data suggest that ketone bodies induced by SGLT2 inhibitors may exert anti-senescent effects. In a different study, the effects of dapagliflozin, metformin and combination treatment on renal senescence were compared in the senescence-accelerated mouse prone 8 (SAMP8) model, which mimics the natural aging process in the kidney. Regardless of diabetes, dapagliflozin was shown to have a greater anti-senescence effect than metformin, and it was concluded that combination therapy may be more effective in renal senescence^129^. In a separate study, 4 months of dapagliflozin treatment in SAMP8 mice resulted in a reduction in renal senescence markers and SASP proteins (including IL-6, TGF-β, IL-1β). The anti-senescence role of dapagliflozin was related to the downregulation of LTBP2 (latent transforming growth factor-beta binding protein 2)^130^. The effects of SGLT2 inhibitors on kidney aging, senescence and the molecular pathways involved are discussed in detail elsewhere^131^.

### β-cell senescence

Type 2 diabetes is associated with insulin resistance and β-cell damage^132^. On the other hand, β-cell senescence has also been reported to contribute to the development of type 2 diabetes in both humans and mice^40^. In a study comparing the metabolic effects of dapagliflozin and lobeglitazone, an insulin-sensitising thiazolidinedione, in HFD induced obese mice, the senescence marker p16^Ink4a^ was found to be increased in the pancreas of HFD-fed obese mice. When combined with lobeglitazone, dapagliflozin significantly reduced p16^Ink4a^-positive cells. The researchers suggest that thiazolidinediones and SGLT2 inhibitors may improve β-cell function and prevent β-cell senescence in the long term^133^.

### Stem and progenitor cell aging

Growing evidence indicates that the regenerative potential of stem and progenitor cells declines with age; accordingly, aged-senescent stem/progenitor cells contribute to defects in tissue repair, leading to organ dysfunction and disease^134–136^. In this sense, targeting tissue-specific senescent stem/progenitor cells may prevent the detrimental effects of aging^135^. To the best of our knowledge, there are not many studies exploring the relationship between SGLT2 inhibitors and stem/progenitor cell senescence. However, Chi et al. recently reported that in human bone marrow mesenchymal stem cells (MSCs), empagliflozin treatment resulted in increased cell proliferation and migration, as well as inhibition of senescence by reducing the number of SA-β-gal positive cells and p21 protein expression. The study also demonstrated an improvement in angiogenesis and cardiac function when extracellular vesicles isolated from MSCs pre-treated with empagliflozin were administered to rats with a myocardial infarction^137^. On the other hand, a clinical study involving patients with type 2 diabetes indicated that SGLT2 inhibitors did not improve the levels of circulating and endothelial progenitor cells despite their cardiovascular protective effects^138^. Conversely, Hess et al.^139^ found that empagliflozin increased circulating pro-angiogenic progenitor cells in type 2 diabetic patients, suggesting that empagliflozin may improve vascular health by ameliorating the depletion of regenerative cells caused by type 2 diabetes. In addition, empagliflozin reduced the number of pro-inflammatory granulocyte precursor cells with high aldehyde dehydrogenase activity (ALDH^hi^), improving vascular repair capacity through decreased oxidative stress^140^. Interestingly, a recent study reported that empagliflozin ameliorated the radiation-induced damage in hematopoietic stem cells by inhibiting the NADPH oxidase-4/ROS/p-p38 pathway^141^. Considering that radiation can cause senescence in stem/progenitor cells^142^ and activate mTOR signalling^143^, this study suggests that SGLT2 inhibitors may have anti-senescence effects on stem/progenitor cells.

### Adipose tissue and liver

Diabetic db/db mice, a model of type 2 diabetes, that were fed a HFD and treated with an SGLT2 inhibitor, TA-1887 for 4 months exhibited reduced inflammation, oxidative stress, and cellular senescence, especially in the visceral white adipose tissue^60^. Moreover, in epididymal white adipose tissue of mice treated with TA-1887, the mRNA expression of inflammatory and senescence mediators such as IL-6, IL-1b, MCP1, and p16^INK4a^ were significantly reduced relative to the untreated diabetic db/db mice. However, the expression of p21 and p16 in the aorta did not differ between diabetic and diabetic-treated animals^60^. Another study reported that empagliflozin treatment downregulated the mRNA expression of p21^Waf1^ in the liver and white adipose tissue of animals with metabolic syndrome. Additionally, empagliflozin treatment reversed the increased SA-β-gal activity and p21 mRNA levels in HepG2 hepatocyte cells exposed to high glucose^144^. Long et al.^61^ also investigated the effects of empagliflozin treatment on liver senescence in naturally aged male mice. Their findings revealed that empagliflozin treatment led to a reduction in p16 and p21, as well as in fibrosis-related proteins α-SMA, COL1A1, and TGF-β1. Furthermore, the study demonstrated that empagliflozin treatment resulted in the activation of PI3K/AKT, AMPK/SIRT1, and the downregulation of NF-κB in senescent livers^61^. Finally, a recent study by Katsuumi et al.^34^ indicated that administering canagliflozin to obese mice fed a HFD resulted in a reduction of senescent cells in visceral adipose tissue.

### Skeletal muscle

Sarcopenia is the age-related loss of skeletal muscle mass and/or function, due to a reduction of protein synthesis and a simultaneous increase in muscle protein degradation, as well as deterioration in muscle regeneration. In a naturally aging mouse model, which showed decreased skeletal muscle function, increased fibrosis, downregulated AMPKα expression and upregulated MMP9/TGFβ1/Smad signalling pathways; this trend was reversed by empagliflozin treatment. At the cellular level, empagliflozin inhibited fibrosis through AMPKα- dependent TFG-β1/Smad signalling as well as the migratory ability of the skeletal muscle fibroblasts stimulated by TFG-β1^145^.

### SGLT2 inhibitors and SIRT1 in senescence

SIRT1, a NAD^+^-dependent deacetylase, regulates various processes, including cellular senescence and aging^146,147^. Studies have reported that SIRT1 expression declines with age^146,148^, and SIRT1 exerts protective effects against age-related diseases^149^. Therefore, SIRT1 appears to be a potential therapeutic target for age-related diseases^150^. SGLT2 inhibitors have been reported to reduce oxidative stress by activating SIRT1/AMPK signalling^78,151^. Furthermore, a bioinformatic study that identified genes interacting with SGLT2 found that SIRT1-SGLT2 was among the interactions involving autophagy, oxidative stress, aging/senescence, inflammation, and AMPK pathways^152^.

A study by Tai et al.^77^ demonstrated that dapagliflozin has protective effects on endothelial cell senescence in diabetic mice, which are mediated by SIRT1. It was found that dapagliflozin improved the decreased SIRT1 expression in senescent endothelial cells induced by high glucose. Additionally, the anti-senescence and ROS-lowering effect of dapagliflozin in senescent endothelial cells was reduced by a SIRT1 inhibitor^74,77^. In a similar study, senescence was induced in HUVECs through H_2_O_2_. Dapagliflozin inhibited senescence evidenced by decreased SA-β-gal activity, p21 and p53 expression. Furthermore, dapagliflozin treatment improved eNOS phosphorylation and SIRT1 expression^74^.

Another study developed an aging model in C57BL/6 mice injected with D-gal to examine the cerebral protective activity of dapagliflozin. Dapagliflozin not only improved the learning and memory skills of aged mice but also significantly increased the activity of the antioxidant enzyme SOD, which was decreased in the brain tissue of aged mice. In addition, dapagliflozin treatment increased the protein expression of SIRT1 and PGC-1α, which also decreased in the brain tissue of aged mice^150^. In a separate study examining the effects of empagliflozin on D-gal-induced renal senescence in C57BL/6J mice, SA-β-gal staining and aging-related proteins p16, LCN2, and TNF-α increased, while SIRT1 expression decreased in the kidneys of D-gal-treated mice. Empagliflozin treatment partially corrected these changes and ameliorated D-gal-induced oxidative stress^153^. Similarly, empagliflozin had comparable effects on senescent HK2 proximal tubule epithelial cells exposed to high glucose. However, the beneficial effects of empagliflozin were limited when cells exposed to high glucose were given a SIRT1 inhibitor. It was concluded that the anti-aging effects of empagliflozin may be attributed to a reduction in SIRT1-mediated oxidative stress^153^.

## Future perspectives

SGLT2 inhibitors, with their anti-inflammatory, anti-oxidant and anti-senescence effects, are emerging as a potential therapy for the prevention of age-related diseases, including cardiovascular, chronic kidney diseases and type 2 diabetes. Calorie restriction is also reported to prolong life by reducing the risk of age-related diseases^154^. The antidiabetic drugs metformin^155^ and acarbose^156^, which mimic the effects of calorie restriction, have been reported to improve healthspan and longevity in mice. Furthermore, it has been hypothesised that SGLT2 inhibitors may have comparable effects to calorie restriction, potentially resulting in an anti-aging effect^62^. Indeed, treatment with canagliflozin^59^ and empagliflozin^61^ were found to extend the lifespan of male mice. However, a recently published study by the same researchers revealed that canagliflozin extended the lifespan of male mice, even when initiated at 16 months of age, but led to a significant reduction in the lifespan of female mice^157^. The observed discrepancy was attributed to the influence of sex-specific differences.

When comparing SGLT2 inhibitors and metformin in terms of their anti-aging effects, it was noted that both drugs have anti-inflammatory properties and can modulate pathways associated with aging (SIRT1, AMPK, p38MAPK). However, it may be the case that SGLT2 inhibitors offer superior benefits over metformin due to their pleiotropic effects^35^. Additionally, another study has demonstrated the neuroprotective effects of canagliflozin on the aging brain^158^, demonstrating SGLT2 inhibitors therapeutic potential for age-related brain diseases^159^. Furthermore, canagliflozin treatment has been demonstrated to delay the onset of premature aging development in a mice model of zinc metalloproteinase Ste24 homologue (Zmpste24) knockout, which exhibits the features of Hutchinson–Gilford progeria syndrome^34^. These findings suggest that canagliflozin may represent a novel approach for the treatment of age-related diseases.

SGLT2 inhibitors show great promise in treating and preventing age-related diseases, especially cardiovascular disease and type 2 diabetes. They exhibit a pleiotropic mechanism of action, specifically targeting senescent cells and modulating several signalling pathways (i.e. SIRT1/AMPK) involved in aging. A recent study by Katsuumi et al.^34^ revealed that in a mouse model of obesity induced by HFD, canagliflozin eliminated the burden of senescent cells, suggesting a potential senolytic effect of the drug. Moreover, this effect was independent of glucose normalisation, by insulin treatment, which had no effect on reducing the senescent cell burden due to HFD. Canagliflozin has been shown to increase a metabolite, 5-aminoimidazole-4-carboxamide-1-β-d-ribofuranoside, which activates AMPK; and AMPK has been shown to modulate programmed cell death-ligand 1 (PD-L1) expression^160^, an immune checkpoint molecule and known to be expressed in senescent cells^161^. Canagliflozin led to a decrease in the number of PD-L1 positive senescent cells in visceral adipose tissue of HFD-fed mice and this effect was inhibited by administration of an additional AMPK inhibitor treatment^161^. On the other hand, administration of a CD3-neutralising antibody to HFD-fed mice attenuated the reduction of SA-β-gal activity by canagliflozin suggesting that the senolytic effect of canagliflozin may be attributable to T cell activation^34^. In contrast, recent research by Jenkins et al.^162^ has demonstrated that canagliflozin, but not dapagliflozin, impedes human T cell effector function in vitro. The study revealed that canagliflozin inhibits T cell receptor signalling, resulting in impaired mTORC1 and ERK activity^162^. On the other hand, metformin, which also activates AMPK, has been shown to phosphorylate serine 195 of PD-L1 and induce abnormal glycosylation, leading to degradation of PD-L1 via endoplasmic reticulum-associated protein degradation^160^. Consequently, a comparable mechanism may be implicated in the suppression of PD-L1 observed with canagliflozin treatment. Further exploration of this hypothesis could provide valuable insights into the underlying mechanisms of SGLT2 inhibitors^163^.

Despite the encouraging outcomes observed in experimental studies, it is acknowledged that further clinical investigations must be conducted to establish the anti-aging effects of SGLT2 inhibitors and their usage as senotherapeutic agents. As a senomorphic agent, metformin has been demonstrated to modulate the SASP^164,165^. Thus, several clinical trials are being carried out to evaluate the effects of metformin on age-related diseases, with a particular focus on cardiovascular disease, and its potential anti-aging properties^166^. One such trial is the 6-year Targeting Aging with Metformin (TAME) Trial, which has been designed to include 3000 non-diabetic individuals aged 65–79 years across the USA^167^. The primary objective of the study is to assess the impact of metformin on the development or progression of age-related chronic diseases, including heart disease, cancer, and dementia. The TAME study is valuable because it was designed to evaluate whether metformin has anti-senescence effects beyond its anti-diabetic properties and if successful, will provide evidence that senotherapeutics can be used to delay the onset of aging and age-related disease.

Similar to the TAME study, determining whether SGLT2 inhibitors improve age-related phenotypes and healthspan in non-diabetic patients beyond their primary function of glucose lowering is an important consideration for designing trials for SGLT2 inhibitors. In this particular context, the impact of SGLT2 inhibitors was examined in patients aged ≥75 years who had been diagnosed with heart failure with reduced ejection fraction (HFrEF) in a retrospective observational study. The results of the study indicated that SGLT2 inhibitors were associated with a reduced rate of all-cause mortality and could potentially enhance the prognosis of older patients suffering from HFrEF^168^. On the other hand, elderly patients may demonstrate heightened intolerance and an increased prevalence of comorbidities, which can limit the usage of novel therapeutic interventions. Indeed, the CANVAS trial has demonstrated that canagliflozin treatment has the potential to increase the risk of bone fracture^14^; this effect, however, has not been consistently observed with empagliflozin^11^ or dapagliflozin^15^. Therefore, it is important to evaluate the potential risks associated with SGLT2 inhibitor use in older patients. Finally, a significant limitation of current research is the focus on male mice in pre-clinical studies of SGLT2 inhibitors and anti-senescence, which raises questions about the generalisability of results to human studies, as it is possible that the results may differ in females^157^. This is especially pertinent considering the sex-differences in terms of myocardial and vascular aging and function in both mice and humans^169,170^.

**Fig. 4:**
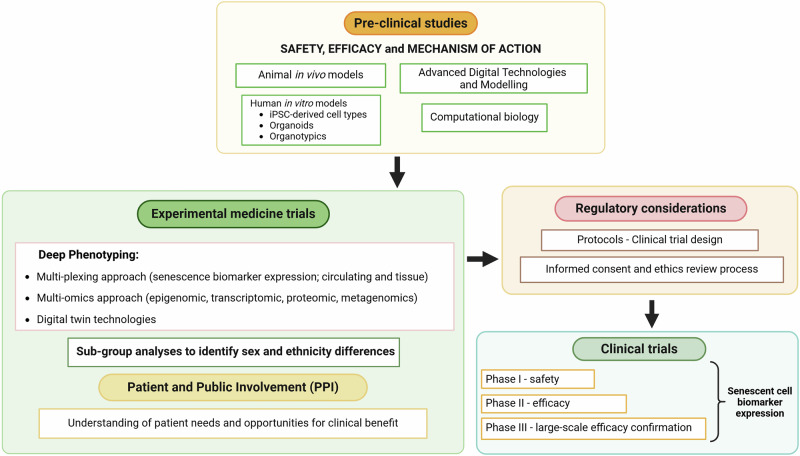
Roadmap for translating findings of pre-clinical and experimental medicine studies into robust human clinical trials. Generated with BioRender (https://biorender.com/).

Future research should elucidate the connection between SGLT2 inhibitors and senescence-associated diseases, incorporating pre-clinical studies conducted in animal models in vivo and human cell in vitro models (iPSC-derived cell types, organoids, organotypic culture) to determine safety, efficacy and mechanism of action. As senescent cell types from different organs/tissues can be phenotypically distinctive and cell-to-cell interaction can be organ/tissue specific, it is vital that the anti-senescence effects of SGLT2 inhibitors is elucidated in the many different cell types (i.e. cardiomyocytes, vasculature, stromal and progenitor cells) that make up the organ. Alongside, advanced digital technologies and computer modelling can simulate organs, such as the heart or kidney, to disease and therapies. These findings would be the first step in creating a roadmap for translating findings into human experimental and clinical trials (Fig. 4). Experimental medicine studies should carry out deep phenotyping utilising multiplexing and multi-omics approaches to interrogate the effects of SGLT2 inhibitors (Fig. 4). Utilising multiplex technologies will determine the level of circulating aging and senescence biomarker expression from blood (SASP factors and immune cell senescence), urine, and saliva. Skin and fat biopsies can also determine change in senescent cell burden. By employing multi-omics (epigenomic, transcriptomic, proteomic, metagenomics) analyses on skin, fat, muscle and fecal samples and using integrated analysis and modelling approaches will ascertain the interplay of multiple biological systems to SGLT2 inhibitors and their role as senotherapeutics. Applying digital twin technologies to simulate the effects of SGLT2 inhibitors, will determine how decreased senescent burden could impact individual organs and systems. Importantly, experimental studies should incorporate a sub-group analyses design to identify sex and ethnicity differences. The cost and capacity to undertake such deep phenotyping approaches in all experimental studies is unrealistic. However, when testing senotherapeutics the assessment of senescent cell burden by circulating and/or tissue senescence biomarker expression should be carried out at the very least. Altogether these data will inform the design and development of robust Phase I/II/III clinical trials to test the therapeutic effect of senotherapeutics, such as SGLT2 inhibitors, in senescence-associated and age-related diseases.

In conclusion, the present findings indicate the clinical potential of SGLT2 inhibition in preventing or delaying age-related diseases through a plethora of mechanisms. Thus, further studies are required to elucidate the efficacy and safety of SGLT2 inhibitors as a senotherapeutic for age-related pathologies.

## Supplementary information

Video of beating iPSC-derived cardiomyocytes
